# A Plant-Produced Antigen Elicits Potent Immune Responses against West Nile Virus in Mice

**DOI:** 10.1155/2014/952865

**Published:** 2014-04-03

**Authors:** Junyun He, Li Peng, Huafang Lai, Jonathan Hurtado, Jake Stahnke, Qiang Chen

**Affiliations:** ^1^The Biodesign Institute, Arizona State University, 1001 S. McAllister Avenue, Tempe, AZ 85287, USA; ^2^School of Life Sciences, Arizona State University, Tempe, AZ 85287, USA

## Abstract

We described the rapid production of the domain III (DIII) of the envelope (E) protein in plants as a vaccine candidate for West Nile Virus (WNV). Using various combinations of vector modules of a deconstructed viral vector expression system, DIII was produced in three subcellular compartments in leaves of *Nicotiana benthamiana* by transient expression. DIII expressed at much higher levels when targeted to the endoplasmic reticulum (ER) than that targeted to the chloroplast or the cytosol, with accumulation level up to 73 **μ**g DIII per gram of leaf fresh weight within 4 days after infiltration. Plant ER-derived DIII was soluble and readily purified to > 95% homogeneity without the time-consuming process of denaturing and refolding. Further analysis revealed that plant-produced DIII was processed properly and demonstrated specific binding to an anti-DIII monoclonal antibody that recognizes a conformational epitope. Furthermore, subcutaneous immunization of mice with 5 and 25 **μ**g of purified DIII elicited a potent systemic response. This study provided the proof of principle for rapidly producing immunogenic vaccine candidates against WNV in plants with low cost and scalability.

## 1. Introduction

West Nile Virus (WNV) belongs to the* Flavivirus* genus of the Flaviviridae family. It is a positive-stranded, enveloped RNA virus that infects the central nervous system (CNS) of humans and animals. Once a disease that was restricted to Old World countries, it entered into the Western hemisphere through New York City in 1999 and has now spread across the United States (US), Canada, the Caribbean region, and Latin America [1]. The outbreaks of WNV have become more frequent and severe in recent years with 2012 as the deadliest yet with 286 fatalities in the US [1]. WNV infection causes fever that can progress to life-threatening neurological diseases. The most vulnerable human population for developing encephalitis, meningitis, long-term morbidity, and death includes the elderly and immunocompromised individuals [2]. Recent studies also identified genetic factors associated with susceptibility to the disease [3, 4]. Currently, no vaccine or therapeutic agent has been approved for human application. The threat of global WNV epidemics and the lack of effective treatment warrant the development of vaccines and production platforms that can quickly bring them to market at low cost.

The WNV Envelope (E) glycoprotein mediates viral binding to cellular receptors and is essential for the subsequent membrane fusion [5]. It is also a major target of host antibody responses [5]. Studies have shown that WNV E shares a three-domain architecture with E proteins of dengue and tick-borne encephalitis viruses [6]. The domain III (DIII) of WNV E protein contains the cellular receptor-binding motifs and, importantly, the majority of the neutralizing epitopes that induce strong host antibody responses and/or protective immunity are mapped to this domain [7]. As a result, DIII has been targeted as a WNV vaccine candidate [8]. Insect cell and bacterial cultures have been explored to express the WNV DIII protein [9, 10]. However, these culture systems are challenged by their limited scalability for large-scale protein production. Moreover, DIII expression in bacterial cultures often leads to the formation of inclusion bodies, which requires a cumbersome solubilization and refolding process to yield a recombinant DIII protein that resembles its native structure [10].

Expression systems based on plants may provide solutions to overcome these challenges, because they provide highly scalable production of recombinant proteins at low cost and have a low risk of introducing adventitious human or animal viruses or prions [11, 12]. Stable transgenic plants were first explored to produce subunit vaccine proteins. While feasible, the low protein yield and the long time period are required for generating and selecting transgenic lines hinder a broad application of this strategy [13]. Recently, transient expression systems based on plant virus have been developed to address these challenges. While the infectivity of plant viruses has been eliminated through viral “deconstruction,” these vectors still retain the robustness of the original plant virus in replication, transcription, or translation [14]. Thus, deconstructed plant viral vectors promote high-level production of recombinant protein within 1 to 2 weeks of vector delivery [14–16]. The MagnICON system is a popular example of these vectors based on* in planta* assembly of replication-competent tobacco mosaic virus (TMV) and potato virus X (PVX) genomes from separate provector cDNA modules [17, 18]. The 5′ module carries the viral RNA dependent RNA polymerase and the movement protein (MP), and the 3′ module contains the transgene and the 3′ untranslated region (UTR).* A. tumefaciens* strains harboring the two modules are mixed together and coinfiltrated into plant cells along with a third construct that produces a recombination integrase. Once expressed, the integrase assembles the 5′ and 3′modules into a replication-competent TMV or PVX genome under the control of a plant promoter [18, 19]. This assembled DNA construct is then transcribed and spliced to generate a functional infective replicon. Geminiviral expression system is another example: a DNA replicon system derived from the bean yellow dwarf virus (BeYDV) [20, 21]. Another interesting example is an expression vector system that is based on the 5′ and 3′-untranslated region of Cowpea mosaic virus (CPMV) RNA-2. This vector system does not require viral replication yet allows high-level accumulation of recombinant proteins in plants [22]. Thus, these plant transient expression systems combine the advantages of speed and flexibility of bacterial expression systems and the post-translational protein modification capability and high-yield of mammalian cell cultures. As a result of this development, a variety of protein vaccine candidates have been produced in plants [11, 12, 23–26]. The immunogenicity of a plant-produced vaccine candidate against WNV has not been described.

Here, we described the rapid production of the WNV DIII in* Nicotiana benthamiana* plants using the TMV-based vectors of the MagnICON system. We demonstrated that DIII can be expressed in three subcellular compartments of the plant cell including endoplasmic reticulum (ER), chloroplast, and cytosol, with the highest accumulation level in ER within 4 days after infiltration. Plant ER-derived DIII was soluble and was readily purified to >95% homogeneity. Further analysis revealed that plant-produced DIII was folded properly as it exhibited specific binding to a monoclonal antibody that recognizes a large conformational epitope on WNV DIII. The immunogenicity of plant-derived DIII was demonstrated in mice as subcutaneous immunization elicited a potent systemic response.

## 2. Results

### 2.1. Expression of WNV E DIII in ER, Chloroplast, and Cytosol of* N. benthamiana* Leaves

**Figure 1:**
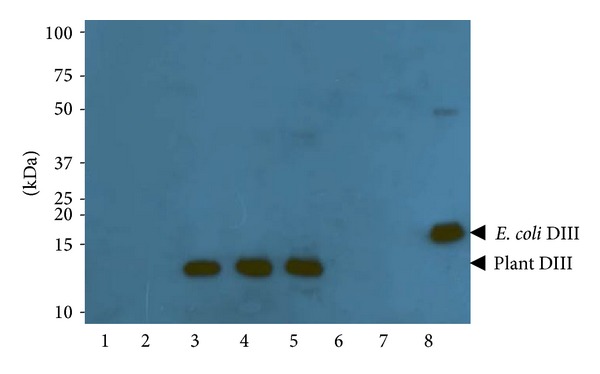
Western blot analysis of DIII expression in* N. benthamiana*. DIII was extracted from* N. benthamiana* leaves and separated on 15% SDS-PAGE gels and blotted onto PVDF membranes. MAb hE16 and a goat anti-human kappa chain antibody were incubated with the membranes sequentially to detect DIII. Lane 1: protein sample extracted from uninfiltrated leaves as a negative control; Lanes 2–5: sample collected 2, 3, 4, and 5 dpi from leaves infiltrated with ER-targeted DIII construct; Lane 6: sample collected 5 dpi from leaves infiltrated with chloroplast-targeted DIII construct; Lane 7: sample collected 5 dpi from cytosol-targeted DIII leaves; Lane 8:* E. coli*-produced DIII as a positive control.

**Figure 2:**
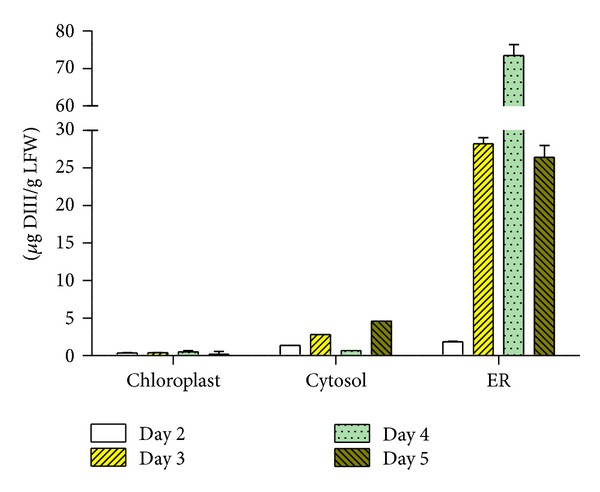
Temporal expression patterns of DIII in chloroplast, cytosol, and ER. Total protein from plant leaves infiltrated with chloroplast, cytosol, or ER-targeted DIII construct was extracted on 2–5 dpi and analyzed by an ELISA with mAb hE16 which recognizes a conformational epitope on DIII and a polyclonal anti-DIII antibody. Mean ± SD of samples from several independent experiments are presented.

To demonstrate the feasibility of using plants to produce a candidate vaccine for WNV, we first determined what subcellular compartment was optimal for DIII accumulation.* Agrobacterium tumefaciens* strain containing the 3′ DIII construct module was codelivered into* N. benthamiana* leaves along with the 5′ module and an integrase construct through agroinfiltration [27, 28]. Three different 5′ modules were specifically chosen to target DIII into ER, chloroplast, or the cytosol [24]. Leaf necrosis was observed in the infiltrated area 4 or 5 days post infiltration (dpi) in plants for all constructs, with cytosol-targeted construct causing the most severe symptoms (data not shown). By 6 dpi, necrosis was too extensive to recover significant amounts of live tissue from the infiltrated leaf area. As a result, DIII expression was examined between 2 and 5 dpi by Western blotting. For the construct targeted to accumulate DIII in ER, a positive band with the predicted molecular weight for DIII (13.5 kDa) was detected on Western blot starting 3 dpi (Figure 1, Lanes 3–5). In contrast, no positive band was detected for chloroplast or cytosol-targeted DIII construct even on 5 dpi (Figure 1, Lanes 6 and 7). An* E. coli*-produced DIII was used as a positive control and, as expected, it was detected as a positive band on the Western blot (Figure 1, Lane 8). The* E. coli*-produced DIII appeared to be larger than that from plants (16.9 kDa), because it contained multiple polypeptide tags from the bacterial expression vector pET28a (EMD Milipore). The lack of positive band in the negative control leaf samples (Figure 1, Lane 1) confirmed the specificity of the DIII band. The expression of DIII was quantified by a sandwich ELISA using two WNV specific antibodies (Figure 2). In leaves that DIII was targeted to the cytosol or chloroplast, the maximal levels of accumulation are below 1.16 *μ*g of DIII per gram of leaf fresh weight (LFW) or 0.01% of total soluble protein (TSP), confirming the result of Western blotting. The ER-targeted DIII reached the highest level of production at 4 dpi, with an average accumulation of 73 *μ*g/g LFW or 0.63% TSP, approximately ~63 times more than that in cytosol or chloroplast (Figure 2).

### 2.2. Purification of DIII from* N. benthamiana* Plants

**Figure 3:**
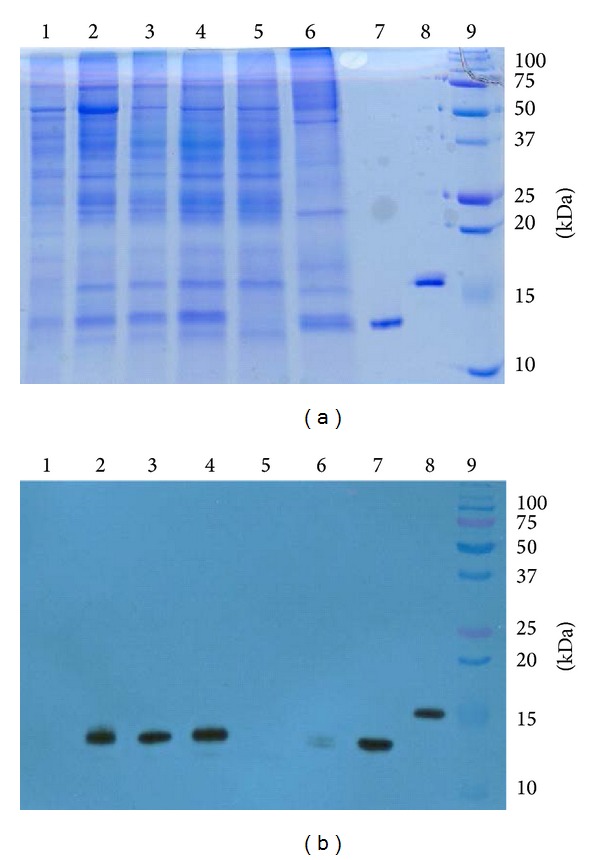
Purification of DIII from* N. benthamiana* leaves. DIII was purified from leaves infiltrated with ER-targeted DIII construct and analyzed on 15% SDS-PAGE gels and either visualized with Coomassie blue stain (a) or transferred to a PVDF membranes followed by Western analysis with hE16 (b). Lane 1: pH 5.0 precipitation pellet; Lane 2: total extracted protein; Lane 3: pH 5.0 supernatant; Lane 4: Ni IMAC loading; Lane 5: Ni IMAC flow through; Lane 6: Ni IMAC wash; Lane 7: Ni IMAC elute; Lane 8:* E. coli*-produced DIII; Lane 9: molecular weight marker.

The availability of an efficient purification scheme is another essential component for plant-derived DIII to become a viable WNV vaccine candidate. Since DIII was tagged with a His_6_ tag, we developed a two-step purification procedure based on acid precipitation and immobilized metal ion affinity chromatography (IMAC). Samples from various purification steps were analyzed by Coomassie blue staining analysis of SDS-PAGE (Figure 3(a)) and Western blot analysis (Figure 3(b)). Interestingly, DIII was efficiently extracted in the soluble protein fraction of plant leaves (Figures 3(a) and 3(b), Lane 2), in contrast to the insoluble inclusion body in* E. coli* [29]. Precipitation with low pH (5.0) removed a large proportion of endogenous plant proteins including the most abundant host protein, the photosynthetic enzyme RuBisCo (Figure 3(a), Lane 1), while leaving DIII in the supernatant (Figure 3(b), Lanes 1 and 3). The pH adjustment from pH 5.0 to pH 8.0, which was required for the binding of DIII to the nickel (Ni) IMAC resin, did not cause any significant change in protein profile (Figures 3(a) and 3(b), Lane 4). Ni IMAC efficiently removed the remaining plant host proteins (Figure 3(a), Lanes 5 and 6) and enriched DIII to greater than 95% purity (Figures 3(a) and 3(b), Lane 7). A faint reactive band was detectable in fractions of total soluble protein, pH 5.0 precipitation, and IMAC flow through (Figure 3(b), Lanes 2–5), suggesting a minor DIII degradation product. Only the intact DIII band with the predicted molecular mass was detected in the purified DIII fraction. Approximately 3.2 mg of purified DIII was obtained from 100 g LFW. These results demonstrated that not only can DIII be rapidly produced in plants, but also isolated and purified to high homogeneity using a scalable purification method.

### 2.3. Plant-Derived DIII Is Specifically Recognized by a Neutralizing Monoclonal Antibody against WNV DIII

**Figure 4:**
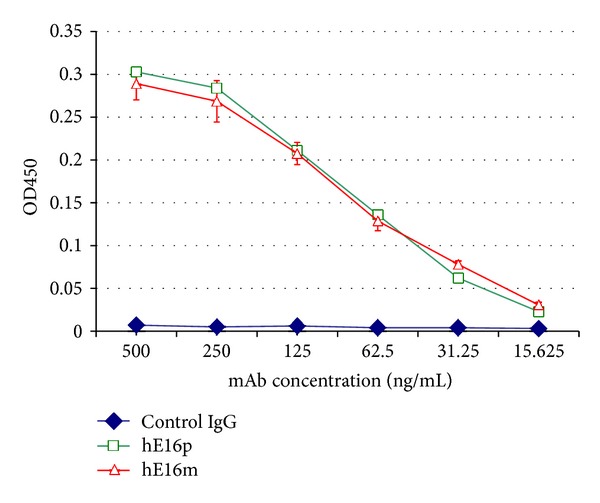
Specific binding ELISA of hE16 to plant-derived DIII. Serial dilutions of hE16 purified from mammalian or plant cells were incubated in sample wells coated with plant-produced WNV DIII and detected with an HRP-conjugated anti-human gamma antibody. A commercial generic human IgG was used as a negative control. Mean ± SD of samples from three independent experiments is presented.

To establish a similarity of structural and immunological properties between plant-produced and the native viral DIII, we examined the binding of plant-derived DIII to a monoclonal antibody (mAb) hE16 generated against WNV E. Our previous studies have shown that hE16 not only had potent neutralizing activity, but it also effectively protected mice from a lethal infection of WNV in both prophylactic and postexposure models [30, 31]. Since hE16 binds a conformational epitope that consists of 4 discontinuous secondary structural elements of the native WNV DIII [32], recognition of a recombinant DIII by hE16 will be informative of its proper folding. ELISA results showed that plant-produced DIII demonstrated specific binding to hE16 produced in mammalian cell culture (Figure 4). DIII also specifically bound to a plant-derived hE16 that showed potent therapeutic efficacy in mice (Figure 4) [30]. Similar results were obtained with the sandwich ELISA used for the quantification of DIII in plant extracts (data not shown). These results indicate that plant-produced DIII was folded into a tertiary structure that resembled the native viral DIII on the surface of WNV.

### 2.4. Plant-Produced DIII Elicits Potent Systemic Immune Response in Mice

**Figure 5:**
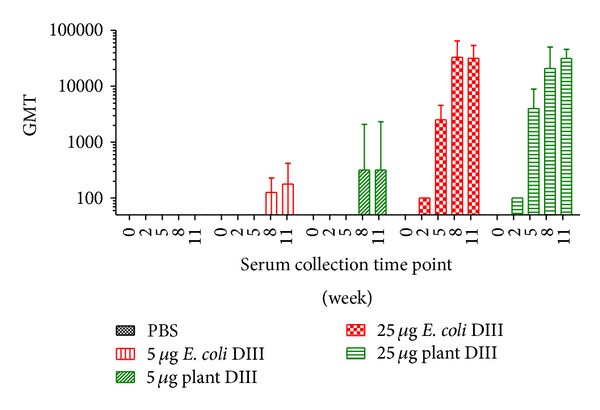
Time course of DIII specific antibody responses in mice upon subcutaneous delivery of plant-derived DIII. BALB/C mice (*n* = 6 per group) were injected on weeks 0, 3, 6, and 9 with the indicated dosage of antigen. Blood samples were collected on the indicated weeks and serum IgG was measured by ELISA. The *y*-axis shows the geometric means titers (GMT) and the error bars show the 95% level of confidence of the mean.

To evaluate the immunogenicity of plant-derived DIII, BALB/c mice were injected subcutaneously with four doses of DIII over an 8-week time period (on days 0, 21, 42, and 63). Two dosages of 5 *μ*g and 25 *μ*g of DIII were tested with alum as adjuvant. Mice were divided into 5 groups (*n* = 6 per group), with group 1 as the negative control group injected with alum + saline (PBS), groups 2 and 3 with plant-derived DIII, and groups 4 and 5 with* E. coli*-produced DIII as a control. Individual serum DIII-specific antibody responses were measured by ELISA and Geometric mean titer (GMT) was calculated for each group at various time points (Figure 5). Samples collected from the control PBS group throughout the entire experiment course and preimmune sera for all groups taken prior to the first immunization (day 0) were negative for the presence of anti-DIII IgG (titer < 10) (Figure 5). All mice in groups immunized with 25 *μ*g of DIII responded after the first administration, while a response was only detectable after the third DIII delivery for mice immunized with the lower dosage (5 *μ*g). This dose-dependent trend was also reflected in the amplitude of the response throughout the various time points of the immunization. For groups receiving DIII, IgG titers increased after each of the first three antigen's delivery and reached its peak at week 8, two weeks after the third immunization. Antibody titers at week 11 (two weeks after the fourth dose) were similar to those of week 8 for all groups except the 5 *μ*g* E. coli*-DIII group (Figure 5). This indicated that the last immunization did not significantly further boost the DIII-specific antibody response, especially in mice that received the higher dosage of DIII. Compared with* E. coli*-produced DIII, plant-derived DIII showed at least equivalent potency (*P* > 0.5) in eliciting humoral response against WNV (Figure 5).

In order to evaluate the Th type of response induced by DIII, antigen-specific IgG subtypes IgG1 and IgG2a were evaluated by ELISA for samples collected at week 11 from mice that were immunized with 25 *μ*g of* E. coli*- or plant-derived DIII. As shown in Table 1, >99% of DIII-specific IgG belonged to the IgG1 subtype, indicating an overwhelmingly Th2-type response stimulated by DIII antigen with alum as the adjuvant.

### 2.5. Characterization of Antiserum against Plant-Derived DIII Antigen

**Figure 6:**
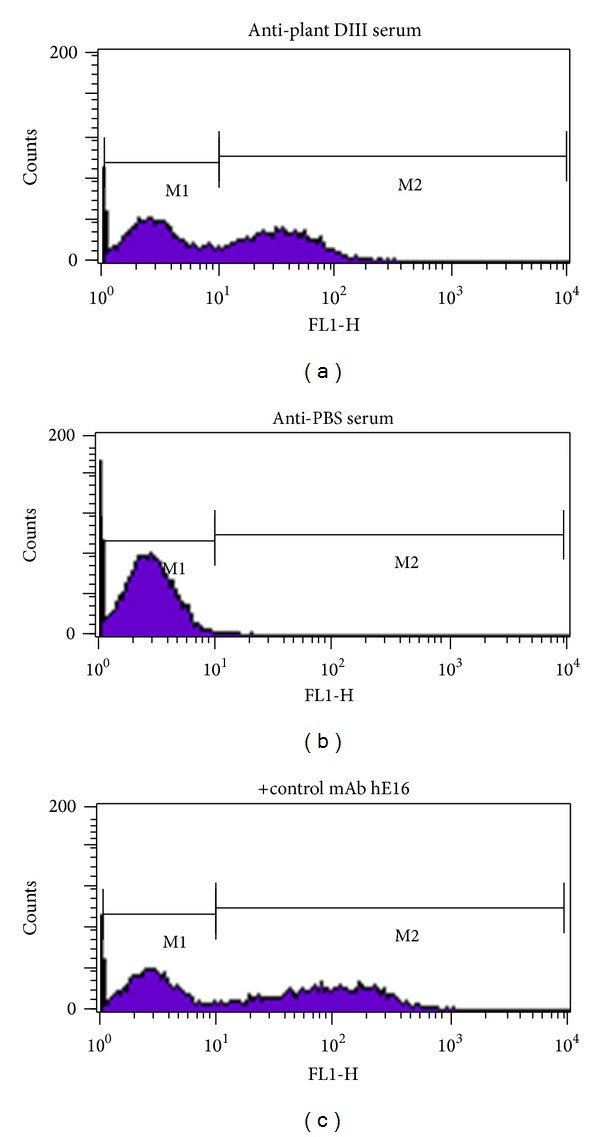
Binding of antibodies in anti-DlII serum to DIII displayed on yeast cell surface. DIII displaying yeast cells were incubated with pooled sera collected on week 11 from mice injected with either 25 *μ*g of plant-produced DIII (a) or PBS (b). hE16 was used as a positive control mAb (c). After incubation, yeast cells were stained with an Alexa Fluor 488-conjugated goat anti-mouse (a and b) or goat anti-human (c) secondary antibody and processed by flow cytometry.

**Figure 7:**
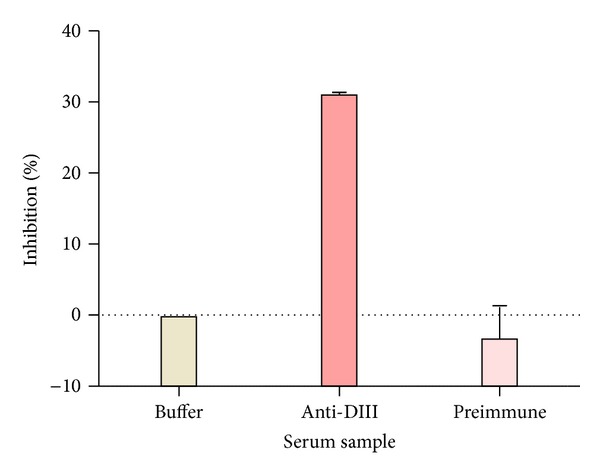
Competitive ELISA of DIII binding by hE16 and antibodies in anti-DIII serum. Plant-derived DIII immobilized in microtiter plate wells was preincubated with 1 : 100 dilution of indicated sera. hE16 was then applied to sample wells to determine its binding to DIII. The inhibition of serum preincubation on the subsequent hE16 binding to DIII is presented as the % of OD_450_ reduction by the preincubation. Mean ± SD of samples from three measurements is presented.

Antisera obtained at week 11 from mice of the 25 *μ*g plant-DIII group were examined in a binding assay with yeast that displayed DIII in its native conformation on its surface. Flow cytometric analysis demonstrated that antibodies in the anti-DIII sera displayed positive binding to DIII on the surface of the yeast (Figure 6(a)). This indicated that anti-DIII sera contained antibodies that can recognize the native viral DIII protein. Similar positive binding was observed for positive control mAb hE16 (Figure 6(c)), but not for equivalent antisera from mice that were immunized with PBS (Figure 6(b)). To investigate if plant-DIII elicited antibodies that bind to the same epitope as the protective mAb hE16, antisera were further analyzed with a competitive ELISA. Results showed that preincubation of DIII with antisera from immunization of plant-derived DIII significantly inhibited its binding to hE16 (Figure 7). No reduction in DIII binding to hE16 was observed when it was preincubated with preimmune serum. This indicated that plant-produced DIII induced the production of anti-DIII IgGs that bind to the same protective epitope as hE16 or at least to epitopes adjacent to that one. This suggested some of the antibodies in the anti-DIII sera were potentially neutralizing and protective.

## 3. Discussion

WNV has caused continuous outbreaks in the US since its introduction in 1999. While the number of cases fluctuated and even dropped from 2008 to 2011, the illusion that its transmission would remain at a low rate quickly evaporated as a large WNV epidemic with high incidence of neurological disease broke out in 2012. WNV was also reported to expand into new geographic areas in Europe and other parts of the world. Therefore, the world may face larger and more severe WNV outbreaks associated with human morbidity and mortality. In the absence of an effective treatment, the need for an effective WNV vaccine is more urgent than ever to halt its expansion and to protect human populations that are vulnerable for developing neurological complications.

Previous studies showed that immunization of DIII produced in* E. coli* or insect cell cultures with CpG oligodeoxynucleotide adjuvant or in fusion with bacterial flagellin elicited WNV-neutralizing antibodies in mice and, in certain instances, protected mice from WNV infection [29, 33, 34]. While encouraging, these expression systems may not be able to provide the scale and robustness for WNV manufacturing, as the global threat of WNV epidemics demands a scalable production platform that can quickly produce large quantities of vaccines at low cost. Moreover, DIII is often recovered in the insoluble inclusion bodies in bacterial cultures, thus requiring a cumbersome solubilization and refolding process to yield DIII proteins that resemble their native conformation [29]. The high level of endotoxins in* E. coli*-based expression system also raises biosafety concerns and demands an expensive process of purification and validation for their removal to ensure the safety of the final product [10].

Here, we demonstrated that a transient plant expression system provided a rapid production of WNV DIII in* N. benthamiana* plants. In contrast to forming insoluble aggregates in* E. coli* cultures, DIII was produced as a soluble protein in plant cells. As a result, it can be directly extracted and purified to >95% homogeneity by a simple and a scalable purification scheme without the time-consuming process of denaturing and refolding. This enhanced the likelihood of producing DIII protein that displays its native conformation. Indeed, plant-derived DIII appeared to fold properly as it was specifically recognized by hE16, a protective anti-WNV mAb that binds a large conformational epitope spanning 4 distinct regions of DIII.

Within the three subcellular compartments we tested, DIII accumulated at much higher levels in ER than in chloroplast and cytosol. The highest expression level was achieved rapidly at 4 dpi, with an average accumulation of approximately 73 *μ*g/g LFW. This level is lower than that of other pharmaceutical proteins we have produced with the MagnICON system [24, 30, 35]. The induction of leaf necrosis by DIII may contribute to the lower expression level as it may shorten the window for accumulation. It is not clear if the observed leaf necrosis is caused by an inherent toxicity of DIII or by the employed overexpression system. To our best knowledge, WNV DIII has not been produced in another plant species or with another plant expression system. We also speculate that the 73 *μ*g/g LFW was a conservative estimate from the early small-scale expression experiments, as we routinely obtained 30–70 *μ*g of purified DIII from 1 g of* N. benthamiana* leaves with 30–50% recovery rate in pilot scale experiments (Chen, unpublished data). The underestimation could be partially attributed to the fact that hE16 was used as a capture antibody in the ELISA, as it only detected fully folded DIII that displayed the specific conformational epitope. Regardless, this expression level of WNV DIII is still the highest compared with other plant-produced* Flavivirus* vaccine proteins, including DIII of dengue virus expressed with a TMV-based vector in tobacco [36]. Since the production of DIII was performed under standard conditions, its accumulation level in plants can be further increased by genetic and environmental optimizations.

Production of DIII by using plant-expression systems may also overcome the challenge of limited scalability and cost issues associated with bacterial and insect cell culture systems. The scalability of both upstream and downstream operations for transient plant expression systems has been recently demonstrated. For example, we used nontransgenic* N. benthamiana* plants for DIII production in this study. As a result, the wild-type plant biomass can be cultivated and produced in large scale with routine agriculture practice without the need to build extraordinarily expensive cell culture facilities [23, 37–39]. We previously demonstrated that commercially produced lettuce could be used as an inexpensive and virtually unlimited source for pharmaceutical protein production [40]. Accordingly, the agroinfiltration process to deliver DIII DNA construct into plant cells has been automated and can be operated in very large scales. For example, several metric tons of* N. benthamiana* plants are regularly agroinfiltrated per hour by using a vacuum infiltration procedure [27, 28]. For downstream processing, our extraction and purification procedure eliminated the hard-to-scale up steps of denaturing and refolding and allowed the recovery of highly purified DIII with a simple two-step procedure of low pH precipitation and IMAC. The scalability of the downstream process, consisting of precipitation and affinity chromatography, has been extensively demonstrated by the pharmaceutical industry and by our studies with other plant-produced biologics [30, 41]. This simple and scalable downstream process from plants will also reduce the costs associated with denaturing and refolding procedures and the overall cost for DIII production. The cost-saving benefit of plant-expression systems was also extensively documented by several case studies.

Our results also indicated that plant-produced DIII showed at least equivalent potency in eliciting humoral response against WNV in mice as* E. coli*-produced DIII. The demonstration of antibodies in anti-plant DIII serum that competed with hE16 for the same DIII epitope indicates the induction of potentially protective antibodies against WNV. It is interesting that both plant- and* E. coli*-produced DIII evoked a Th2-type response with alum as the adjuvant. This is in contrast to a previous report that* E. coli* DIII with CpG adjuvant stimulated a Th1-biased response [33]. This is not totally unexpected, as comparative studies with* Flavivirus* antigens showed that alum tends to induce Th2 type response, while CpG is likely to skew the response toward the Th1 type [42]. Since* E. coli*-produced DIII was shown to be protective in the mouse challenge model [29, 33, 34], the equivalent potency of plant-DIII in generating high IgG titers and the induction of hE16-like antibodies suggest that it is highly likely that plant-DIII will induce protective immunity when a proper adjuvant is used. Overall, the rapidity of DIII expression, the availability of a simple purification scheme, and the low risk of contamination by human pathogen and endotoxin indicate that plants provide a robust and low-cost system for commercial production of subunit vaccines against WNV and other flaviviruses.

## 4. Experimental Procedures

### 4.1. Construction of DIII Expression Vectors

The coding sequence of WNV E DIII (amino acid 296–415, Genbank Acc. number AF196835) was synthesized with optimized* N. benthamiana* codons [43]. An 18 bp sequence coding for the hexa-histidine tag (His_6_) was added to the 3′ terminus of the DIII gene and then cloned into the TMV-based expression vector pIC11599 of the MagnICON system [30, 43]. The MagnICON vectors were chosen because they have been demonstrated to drive high-level accumulation of recombinant proteins in* N. benthamiana* plants [30, 31, 38, 41, 43].

### 4.2. Expression of WNV E DIII in* N. benthamiana* Leaves

Plant expression vectors were transformed into* A. tumefaciens* GV3101 by electroporation as previously described [24].* N. benthamiana* plants were grown and agroinfiltrated or coagroinfiltrated with the GV3101 strain containing the DIII-His_6_ 3′ module (pICH11599-DIII) along with one of its respective 5′ modules (pICH15579 for cytosol targeting, pICH20999 for ER targeting, or pICH20030 for chloroplast targeting) and an integrase construct (pICH14011) as described previously [27, 28, 30, 38, 41].

### 4.3. Extraction and Purification of DIII from* N. benthamiana* Leaves

Agroinfiltrated* N. benthamiana* leaves were harvested 2–5 dpi for evaluating DIII expression. Leaves were harvested 4 dpi for other protein analysis. Leaves were homogenized in extraction buffer (100 mM Tris-HCl, pH 8.0, 150 mM NaCL, 1 mM PMSF, tablet protease inhibitor cocktail (Sigma, Germany) at 1 mL/g LFW). The extract was clarified by centrifugation at 18,000 ×g for 30 min at 4°C. The pH of the clarified extract was adjusted to 5.0 and subjected to centrifugation at 18,000 ×g for 30 min at 4°C. The supernatant was recovered, pH adjusted back to 8.0, and subjected to another centrifugation. The supernatant was then subjected to Ni IMAC on a 4 mL His. Bind column in accordance with the manufacturer's instruction (Millipore, USA). The purified WNV DIII was eluted with imidazole and the eluate was dialyzed against PBS. The purity of DIII was estimated by quantitating Coomassie blue-stained protein bands on SDS-PAGE using a densitometer as described previously [30].

### 4.4. SDS-PAGE, Western Blot, and ELISAs

Samples containing DIII were subjected to 15% SDS-PAGE under reducing (5%* v/vβ*-mercaptoethanol) conditions. Gels were either stained with Coomassie blue or used to transfer proteins onto PVDF membranes (Millipore, USA). Membranes were first incubated with MAb hE16 [30] and then subsequently with a goat anti-human kappa antibody conjugated with horseradish peroxidase (HRP) (Southern Biotech). Specific bindings were detected using an “ECL plus” Western blot detection system (Amersham Biosciences).

The expression of WNV DIII protein in leaves was determined by a sandwich ELISA. Ninety-six well ELISA microtiter plates (Corning Incorporated, USA) were coated at 1 *μ*g/mL hE16 mAb in coating buffer (100 mM Na_2_CO_3_, pH 9.6) overnight at 4°C. After washing three times with PBST (PBS containing 0.1% Tween-20), plates were blocked with blocking buffer (PBS containing 5% milk) and incubated with plant extracts. Purified bacterial WNV DIII was used as a positive control to generate the standard curve. Extracts from uninfiltrated plants were used as a negative control. After washing, the plate was incubated with a rabbit anti-WNV DIII polyclonal antibody [43], followed by an HRP-conjugated goat anti-rabbit IgG (Southern Biotech). The plates were then developed with TMB substrate (KPL Inc). Values from negative control leaves were used as “background” of the assay and were subtracted from the corresponding values obtained from DIII construct-infiltrated leaves.

The hE16 recognition ELISA was performed as described previously [30]. Briefly, purified plant-DIII was immobilized on microtiter plates. After incubation with hE16 purified from mammalian cells or from plants, an HRP-conjugated goat anti-human-gamma HC antibody (Southern Biotech) was used to detect bound antibodies. A generic human IgG (Southern Biotech) was used as a negative control.

The titer of DIII-specific IgG in mouse serum was also determined by an ELISA. Microtiter plates were coated with plant- or* E. coli*-derived DIII, blocked with PBS with 1% bovine serum albumin (BSA), and incubated with a serial dilution of serum. After washing with PBST, the plates were incubated with an HRP-conjugated goat anti-mouse IgG (H + L) (Southern Biotech). After further washing with PBST, the plates were developed with TMB substrate (KPL Inc). Geometric mean titer (GMT) was calculated for each group at various time points and was used to express the titer of the DIII specific IgG.

The ELISA for determining the IgG1 and IgG2a subtypes were performed also on plates coated with plant- or* E. coli*-derived DIII as described above. Serial dilutions of serum were applied to sample wells and incubated for 2 hr at 37°C. After washing with PBST, the plates were incubated with an HRP-conjugated goat anti-mouse IgG1 (Santa Cruz Biotech) or anti-mouse IgG2a (Southern Biotech). In parallel, various dilutions of mouse IgG1 and IgG2a (Southern Biotech) were coated on the same set of plates for generating standard curves. The plates were developed with TMB substrate (KPL Inc.).

A competitive ELISA was also performed on plates coated with DIII purified from plants. After blocking, plates were preincubated with serial dilutions of serum from pooled preimmune serum (Group 3), or pooled serum collected at week 11 (Groups 1 and 3). After thorough washing with PBST, plates were incubated with hE16, subsequently an HRP-conjugated goat anti-human-gamma HC antibody (Southern Biotech), and developed with TMB substrate (KPL Inc). The inhibition of hE16 binding to DIII by preincubation of sera was calculated by (Binding_(no  pre-incubation)_ − Binding_(pre-incubation  with  serum)_)/Binding_(no  pre-incubation)_.

All ELISA measurements were repeated at least three times with each sample in triplicate.

### 4.5. DIII Expression in* E. coli* and Yeast Surface Display

The synthesized DIII coding sequence was cloned into the pET28a bacterial expression plasmid (EMD Milipore) with EcoRI and HindIII sites. DIII was expressed in* E. coli* and purified using an oxidative refolding protocol as described previously [44]. Refolded DIII protein was further purified with a Ni His. Bind IMAC as described for plant-derived DIII.

Yeast expressing WNV DIII was generated and stained with mAbs as described previously [30]. Briefly, yeast cells were first grown to log phase and subsequently induced for DIII expression by an additional 24 h culture in tryptophan-free media containing 2% galactose. The yeast cells were then incubated with pooled mice serum collected in week 11 from the DIII immunization experiments or hE16 mAb as a positive control [30]. Serum from the saline mock-immunized mice was used as a negative control. The yeast cells were stained with a goat anti-mouse or goat anti-human secondary antibody conjugated with Alexa Fluor 488 (Invitrogen). Subsequently, the yeast cells were analyzed on a BD FACSCalibur flow cytometer (Franklin Lakes).

### 4.6. Mouse Immunization

All animal work was approved by the institutional animal care and use committee. Five-week old female BALB/C mice were divided into 5 groups (*n* = 6 per group). Group 1 received saline buffer (PBS) with alum as mock immunized control. Groups 2 and 3 received 5 *μ*g and 25 *μ*g of plant-derived DIII per dosage, respectively. Groups 4 and 5 received 5 *μ*g and 25 *μ*g of* E. coli*-produced DIII per dosage as controls. On day 0, each mouse was injected subcutaneously with 100 *μ*L of material containing saline (Group 1), 5 *μ*g (Groups 2 and 4), or 25 *μ*g (Groups 3 and 5) purified DIII protein in PBS with alum as adjuvant (Sigma, DIII Protein solution: alum volume ratio = 1 : 1). Mice were boosted three times (on days 21, 42, and 63) with the same dosage and immune protocol as in the 1st immunization. Blood samples were collected from the retroorbital vein on day 0 before the immunization (pre-immune sample) and on days 14 (2 week), 35 (5 week), 56 (8 week), and 77 (11 week) after the 1st immunization. Serum was stored at −80°C until usage.

## Figures and Tables

**Table 1:** Anti-DIII IgG subtypes (IgG1 and IgG2a) of pooled serum samples.

	Group 3			Group 5		
	Concentration (*μ*g/mL)	SEM	Subtype/total %	Concentration (*μ*g/mL)	SEM	Subtype/total %
IgG1	506.33	58.00	99.5%	488.00	48.08	99.8%
IgG2a	2.67	0.70	0.5%	0.98	0.44	0.2%

Serum samples collected at week 11 were pooled for each indicated group and analyzed by ELISA for IgG1 and IgG2a antibody concentration. Mean concentration (*μ*g/mL) of the IgG subtype and the standard error of the mean (SEM) from several independent measurements are presented. Group 3: mice received 25 *μ*g per dosage of plant-derived DIII; Group 5: mice received 25 *μ*g per dosage of *E. coli*-derived DIII.
